# Should all electrocardiography be ambulatory?

**DOI:** 10.1007/s12471-023-01804-0

**Published:** 2023-08-15

**Authors:** Joris R. de Groot, Ralf E. Harskamp

**Affiliations:** 1 Department of Cardiology, Heart Centre, Amsterdam Cardiovascular Sciences, Amsterdam University Medical Centres, location Academic Medical Centre/University of Amsterdam, Amsterdam, The Netherlands; 2 Department of General Practice, Amsterdam Public Health research institute and Amsterdam Cardiovascular Sciences, Amsterdam University Medical Centres, location Academic Medical Centre/University of Amsterdam, Amsterdam, The Netherlands

Due to technological advances, electrocardiographic parameters can now be obtained by the lay public without going to a clinic or hospital. Consumer-based wearable devices that allow detection of atrial fibrillation (AF) and other arrhythmias are increasingly being used. Indeed, we have learned that early detection of AF can lead to earlier therapy and prevention of untoward thromboembolic complications. Another lesson learned is that population-based screening with such devices requires a sufficiently high pretest likelihood and a test with sufficient sensitivity and specificity to provide sensible outcomes. An important example in this context is the Apple Heart Study, which provided data on ~ 0.5 million persons who used a smartphone app for AF detection. As participants in that study overall reflected the ‘worried well’, with a very low pretest likelihood of AF, it is not surprising the test characteristics derived from this study were suboptimal [1].

Apart from these considerations, there is a presumption that device-detected AF carries the same stroke risk as clinical AF. However, this is far from certain. We do know that stroke risk is increased in patients with device-detected atrial high rates, but this risk depends, at least in part, on AF duration [2–4]. Yet, it is not said that the same recommendations for oral anticoagulation apply to patients with overt AF and those with device-detected AF only. For example, The LOOP Study, in which implantable loop recorders were implanted in high-risk patients without AF using a population screening setup, demonstrated that device detection of AF led to increased prescription of anticoagulant drugs but not fewer strokes or lower mortality [5]. This is just an example to show that the availability of more data does not necessarily result in more advanced care. It also illustrates that we should be cautious to overgeneralise findings from one population and healthcare setting to another. Finally, ambulatory monitoring carries the risk of severely increasing the workload of healthcare professionals, as recordings acquired outside the hospital require confirmation, if only for legal responsibility reasons.

The algorithms behind those systems usually do not go further than giving the advice to consult a doctor in case of an abnormal recording. This is irrespective of the notion that machine detection of AF is as accurate as human assessment; it is simply the result of an algorithm that cannot take responsibility for clinical care. Moreover, there are also electrocardiographic findings of which we do not directly know the clinical implications, resulting in additional diagnostic work-up, increased healthcare spending and the risk of unjustly turning a healthy person into a patient.

In this issue of the *Netherlands Heart Journal*, Bergeman and colleagues present a proof-of-concept study in 235 patients to quantify QT intervals with a 6-lead mobile electrocardiogram (mECG) device (Kardia Mobile 6L; AliveCor, Mountain View, CA, USA) [6]. For AF detection, this device is more accurate than single-lead devices [7]. The authors provide context for their study by stating that repeating 12-lead ECGs can be cumbersome (particularly in a community-based setting) and mECG devices can potentially fill that gap. They show that only 32% of ambulatory recordings were of good quality, whereas the majority (62%) was of acceptable quality and 7% were of insufficient quality. This observation should be kept in mind when considering implementation into clinical practice. All patients also had a 12-lead ECG taken, and in 98%, there was agreement on the rhythm between the mECG and conventional ECG. Heart rate was consistently higher in the mECG recordings than in the conventional ECG recordings, but the mECG was recorded in sitting position, whereas the 12-lead ECG was taken while the patient was supine. PR interval and QRS duration moderately to strongly correlated between the modalities.

Of the 234 mECG recordings, 33 did not allow for QT determination in lead I and 31 not in lead II; neither lead was suitable in 16 patients (7%) [6]. The QT interval could not be measured from lead I or II in 43 and 3 conventional ECGs, respectively, and in neither lead in one patient. The QT interval was 396 ± 30 and 395 ± 30 ms in lead I of the mECG and 12-lead ECG, respectively. The mean absolute QT interval difference using lead I was 14 ± 13 ms, and 44% of the patients showed a < 10 ms difference in this lead, whereas the mean absolute difference in lead II was 12 ± 9 ms (< 10 ms difference in 55% of subjects), which was similar to a previous QT interval comparison between a single-lead mECG and conventional ECG [8]. In patients with QT prolongation > 480 ms, the sensitivity and specificity for QT prolongation were 80% and 99%, respectively.

The authors rightfully make the point that mECG measurement of the QT interval is feasible [6]. Does that imply that we should now have our patients record their QT interval in an ambulatory manner? We are not convinced. First, the indication for QT interval monitoring is usually a condition or a change in medication invoked by a physician. The responsibility for the safe use of such medications lies with that physician and can, in our opinion, not be delegated to patients. Second, an abnormal finding has immediate consequences that can go as far as patient admission to a clinical ward for telemetric monitoring. Hence, it is not illogical to have patients visit their doctor, who can make an ECG and adapt their therapy or admit them when needed.

An ultimate consequence of the possibility of ambulatory QT measurement is that treatment decisions move to primary care and that general practitioners (GPs) will have to provide care for patients at risk of QT prolongation. It can be questioned whether GPs have the infrastructure to provide such care and whether they are motivated to take that responsibility. Patients taking class 1 and class 3 antiarrhythmic drugs for AF are usually followed up in secondary care [9]. We would argue that the same accounts for QT interval monitoring, either in patients at risk of QT prolongation (i.e. those with long QT syndrome) or in patients on QT-prolonging medication. Whether ambulatory recording of the QT interval facilitates that process remains to be determined. As a minimum, an infrastructure that collects data and provides QT measurement is required. Currently, QT measurement requires human confirmation—even when acquired with a mobile device—, which is a time-consuming and costly endeavour. Perhaps the patient should just come to the clinic to have an ECG taken and speak with their treating cardiologist about the consequences of the QT interval they recorded.
